# The Effect of EEG Neurofeedback Training on Sport Performance: A Systematic Review and Meta‐Analysis

**DOI:** 10.1111/sms.70055

**Published:** 2025-04-24

**Authors:** Chien‐Lin Yu, Ming‐Yang Cheng, Xin An, Ting‐Yu Chueh, Jia‐Hao Wu, Kuo‐Pin Wang, Tsung‐Min Hung

**Affiliations:** ^1^ Department of Physical Education and Sport Sciences National Taiwan Normal University Taipei Taiwan; ^2^ Department of Sport Science Research Taiwan Institute of Sports Science Taipei Taiwan; ^3^ School of Psychology Beijing Sport University Beijing China; ^4^ Master's Program of Transition and Leisure Education for Individuals With Disabilities University of Taipei Taipei City Taiwan; ^5^ Department of Kinesiology Pennsylvania State University University Park Pennsylvania USA; ^6^ Cognitive Interaction Technology Bielefeld University Bielefeld Germany; ^7^ The Master Program of Sport Facility and Health Promotion National Taiwan University Taipei Taiwan; ^8^ Institute for Research Excellence and Learning Science National Taiwan Normal University Taipei Taiwan

**Keywords:** meta‐analysis, motor performance, neurofeedback, review, sport performance

## Abstract

Neurofeedback training (NFT) has emerged as a promising technique for enhancing sports performance by enabling individuals to self‐regulate their neural activity. However, only 53% of the 13 included studies, all of which published before 2021, in the latest meta‐analyses of NFT and motor performance focused on motor performance outcomes. Due to the rapid development of neurofeedback, 8 high‐quality articles were published in 2023–2024 alone. Therefore, there is a need for a new meta‐analysis to update the impact of NFT on sports performance. In this systematic review and meta‐analysis, we have not only reassessed the knowledge of the effect of EEG neurofeedback in motor performance but have also incorporated a standardized methodology, known as the CRED‐nf checklist (Consensus on the reporting and experimental design of clinical and cognitive‐behavioral neurofeedback studies), for methodological evaluation of previous EEG neurofeedback studies. The study protocol was pre‐registered, and a systematic search was conducted across major databases to identify relevant randomized controlled trials. A total of 25 studies were included in the qualitative synthesis, with 21 studies eligible for the meta‐analysis. The meta‐analysis revealed a moderate positive effect of NFT on sport motor tasks, with a Hedges's g of 0.78 with a 95% confidence interval (CI) of 0.49–1.07. Importantly, subgroup analyses showed that studies with higher methodological quality scores, as assessed by the CRED‐nf checklist, had significantly larger effect sizes (Hedges's g = 1.07) compared to lower than median studies (Hedges's g = 0.49). This finding highlights the importance of addressing key methodological gaps, such as reporting on participant strategies, data processing methods, and the relationship between regulation success and behavioral outcomes. In conclusion, NFT showcases a moderate positive impact on sport motor task, particularly when high‐quality methodologies are employed, as assessed by the CRED‐nf checklist, underscoring the importance of rigorous study designs in future research.

## 1 Introduction

Neurofeedback training (NFT), a technique that involves perceiving and learning from one's own brain signals, has become the most widely utilized method within the field of biofeedback. Initially applied to the prevention and rehabilitation of clinical neurological and mental disorders, neurofeedback training has gradually been expanded to enhance the performance of healthy individuals. It is considered a non‐invasive, safe, and potentially effective approach for modulating the brain's neural state [1, 2]. NFT incorporates various signal modalities, including electroencephalogram (EEG) NFT, functional magnetic resonance imaging (fMRI) NFT, and functional near‐infrared spectroscopy (NIRS) NFT. Among the various neuroimaging technologies, EEG NFT is highly valued in sports performance contexts due to its superior temporal resolution, portability, and adaptability to diverse recording scenarios in sports settings [3].

EEG NFT, which has significant potential for improving sports performance, utilizes an adaptive and feature‐based approach to augment athletic performance through the regulation of brain function [4]. Specifically, EEG NFT has demonstrated its efficacy in enhancing cognitive activity, including focus, memory, and motor skills, which are critical for athletic performance. These improvements have also been associated with enhanced sports performance [5, 6]. For example, EEG NFT aimed at enhancing performance in precision sports has been substantiated by a series of studies demonstrating its positive effects on improving sports performance in a variety of motor tasks, such as golf putting and archery [7, 8, 9, 10, 11, 12]. However, recent meta‐analyses showed that so far evidence supporting EEG NFT protocols' effectiveness in changing EEG and improving sports performance was rather weak [13, 14]. The considerable variability in the outcomes of EEG NFT studies can primarily be attributed to significant differences in experimental paradigms and parameters. These variations contribute to conflicting conclusions about EEG NFT's efficacy. For instance, Mirifar et al. [15, 16] highlighted that the quality of these studies is inconsistent; notably, only a few have implemented rigorous double‐blind, placebo‐controlled methodologies, casting doubt on the reliability of EEG NFT's effectiveness. The inconsistent findings across various studies on EEG NFT underscore the complexity of measuring its effectiveness in sports performance enhancement. These discrepancies, ranging from changes in performance without corresponding neural activity to placebo effects [7, 17, 18, 19, 20, 21], highlight the need for more standardized and rigorous research methodologies.

To address the inconsistencies observed in previous neurofeedback studies, the CRED‐nf checklist developed through consensus by Ros et al. [22] could serve as a valuable tool for standardizing methodologies. This CRED‐nf checklist can enhance the reporting and experimental design standards in clinical and cognitive‐behavioral neurofeedback studies. Specifically, this checklist facilitates comprehensive data collection across various study aspects, including pre‐experiment conditions, control groups, feedback specifications, outcome measures, and data storage protocols. Employing this structured approach will sharpen our comprehension of past findings and substantiate the need to evaluate their efficacy. Remarkably, despite its potential utility, this framework has not been systematically applied in reviews or meta‐analyses of EEG NFT in sports performance. Additionally, other review studies have suggested that certain moderators may influence the effectiveness of EEG NFT in enhancing sports performance. These moderators include the following: (1) The participants are either Experts or Novices [23]. Novices and experts may be at different stages of learning, which could influence their performance improvements; (2) integrating NFT into the sport motor task [15, 16, 24]; it could lead to greater transfer of training effects to real‐world performance, as practicing the task concurrently with NFT may strengthen the association between brain regulation and motor execution; (3) number of training sessions [13]; (4) types of athletic performance [25]; (5) quantity of training objectives [25]; and (6) personalized feedback [11]. It may enhance the effectiveness of NFT by optimizing learning and adaptation, whereas general feedback might limit the potential benefits.

To rigorously assess the quality of previous research, our study performs a systematic review and meta‐analysis, which incorporates the CRED‐nf checklist and considers moderators for methodological evaluation. Furthermore, our primary research hypothesis is that studies with higher scores on the CRED‐nf checklist will have a stronger effect of neurofeedback, as high‐quality methodologies are more likely to minimize biases, enhance the reliability of the intervention, and accurately capture the true effects of neurofeedback. Additionally, previous meta‐analyses on neurofeedback have not exclusively focused on sports performance, and many high‐quality articles published in recent years have not been included in the analysis due to the rapid development of neurofeedback. For example, the first meta‐analysis study [14] included 10 studies published before 2015, and a more recent meta‐analysis study [13] included 13 studies published before 2021. To our knowledge, there have been 8 empirical studies published in 2022–2023 alone, meaning that more than half of the high‐quality articles have not been included in the previous meta‐analyses. Furthermore, the proportion of articles focusing on sports performance in the previous two meta‐analyses was only 80% [14] and 53% [13].

Therefore, there is a gap for a new meta‐analysis to identify the impact on sports performance. Not only to update the existing published research but also to focus exclusively on motor performance outcomes. The most important aspect is incorporating the CRED‐nf checklist and moderators for methodological evaluation. We aim to contribute high‐quality insights to the field of EEG NFT in sport and enhance the practical application of EEG NFT research.

## 2 Method

The study protocol for this systematic review and meta‐analysis was registered at PROSPERO (International Prospective Register of Systematic Reviews), with registration number CRD42021238399, and complied with the Preferred Reporting Items for Systematic Reviews and Meta‐Analyses guidelines [26].

### 2.1 Eligibility Criteria

The systematic review was conducted following the Minds Handbook for Clinical Practice Guideline Development 2020 version 3.0 chapter 4 [27]. The PICOS in the current study was as follows: (P) population: healthy human adults (18–64 years); (I) intervention: EEG‐NFT using non‐invasive neuroimaging methods; (C) comparators: any, such as sham neurofeedback, conventional training to improve the target motor skill (e.g., physical training and mental imagery) and condition of no intervention; (O) outcomes: sports motor performance before and after NFT; and (S) study design: a randomized controlled trial (RCT) or a non‐randomized controlled study (NRS).

### 2.2 Search Strategies and Study Selection Process

Three electronic databases, PubMed, Scopus, and Web of Science, were used. All identified articles published prior to January 2025 were included. Search keywords were defined by the research team and were used in each database to identify potential articles with abstract or title, or keywords for review. When searching in PubMed, medical subject heading (MeSH) terms were utilized. The analysis was restricted to the English language and original research articles published in peer‐reviewed journals. Full search terms for all databases are provided in Appendix S1.

### 2.3 Study Selection

Included articles were selected with reference to the PICOS criteria. Titles/abstracts were independently assessed for eligibility in the review by two authors (MYC and XA). Next, the full‐text article review was performed by three authors (CLY, JHW, and MYC). A Microsoft Excel spreadsheet was developed to record eligibility status. Decisions to include or exclude studies were made by consensus. Any disputes were settled by discussion together with additional authors who are experts in the field (CLY, MYC, KPW, and TMH). In addition to the database search, the reference lists of all included studies were checked to identify additional eligible articles [28].

### 2.4 Data Collection Process and Items

**FIGURE 1:**
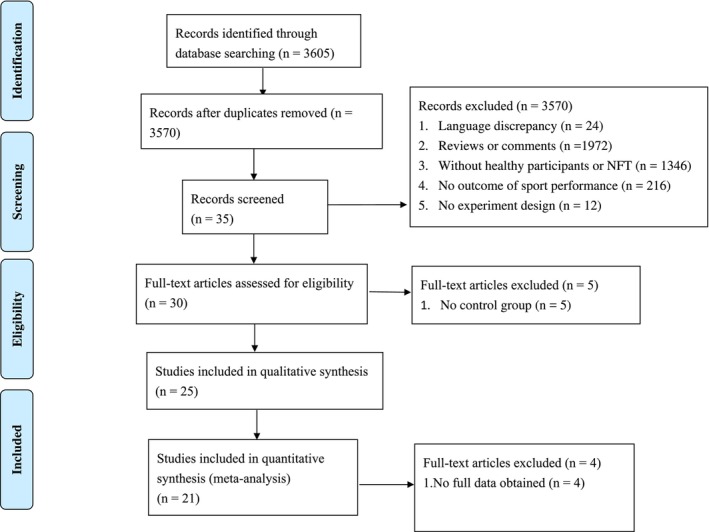
Search processing.

Data extraction was independently conducted by three authors (MYC, JHW, and TYC). In the case of disagreement, discussion ensued until a consensus was reached. Compared to previous meta‐analyses, the distinguishing feature of the current study is the exclusive focus on sports performance. The studies selected for the qualitative synthesis met the following inclusion criteria: (i) implementation of neurofeedback training (NFT); (ii) measurement of motor performance as an outcome, encompassing observable muscular outputs such as reaction time, movement precision and dexterity, and overall body balance; (iii) inclusion of a control group or control condition for the NFT intervention; (iv) publication as an original article in a peer‐reviewed journal, written in English; and (v) study design classified as a randomized controlled trial (RCT) or a non‐randomized controlled study (NRS). Additionally, studies included in the meta‐analysis fulfilled the following criteria: (vi) availability of adequate outcome information to calculate the intervention's effect size and (vii) assessment of the study as having a low or moderate risk of bias (see Figure 1).

### 2.5 Risk of bias in Individual Studies

The quality of the included trials in the systematic review was assessed using the PEDro scale [29]. This scale is recognized for its reliability and validity in assessing research quality [29]. To summarize, the internal validity of the studies was examined across eight criteria: randomization, concealment of allocation, comparability at baseline, accurate reporting and management of exercise intensities, blinding of outcome assessors, completeness of outcome data, adherence to the allocated interventions, analysis of conditions across groups, and the reporting of outcomes with their variability. When available, the PEDro rating and scores were retrieved from the database [30]. If a score was not available from the database, it was independently calculated by three authors (CLY, JHW, and TYC), who had undergone the PEDro training program. Differences were resolved by discussion and adjudicated by another experienced author (TMH) as required. Publication bias was evaluated using Rosenthal's fail‐safe *N* [31] and Egger's regression test [32].

### 2.6 The CRED‐Nf Checklist

Neurofeedback researchers have developed a consensus‐based checklist, known as the CRED‐nf checklist [22], (see Appendix S2), aimed at enhancing the quality of reporting and study design in the fields of clinical and cognitive‐behavioral neurofeedback. This checklist provides detailed guidelines for reporting various aspects of research, including conditions prior to the experiment, the nature of control groups, measures taken for control, specifics of the feedback given, outcomes measured, and how data is stored, as detailed in Appendix S3. A comparative analysis of methodological practices was performed between this study and earlier works. Any disputes were resolved through discussion with additional expert authors (CLY, JHW, and TYC).

### 2.7 Summary Measures

In the conducted meta‐analysis, each study in the articles was treated as an individual experiment. The calculation of effect sizes was based on two methods: (1) the comparison of outcome data between NFT and control conditions, normalized using combined standard deviation (SD) for crossover post‐test trials ([33], 584) and (2) the computation of mean difference scores from before to after NFT versus before to after control conditions, which were then adjusted by an estimated combined SD of change ([33], 166). According to Cohen [34], effect size values of 0.2, 0.5, and 0.8 indicate small, moderate, and large effects, respectively. The data gathered were input into a specially designed spreadsheet that automatically generated the Hedges' *g* to represent the effect size [35]. All information was directly extracted from the published articles, without direct communication with the authors to obtain missing data or further details about their research.

### 2.8 Synthesis of Results

A narrative overview provided in text and Table 1 summarize the study characteristics of all eligible studies. Meta‐analysis was used to calculate summary estimates for the effects of NFT on sports performance using a random model (Comprehensive Meta‐analysis software) when enough studies were included ( > 5; [49]). Evidence of heterogeneity was assessed using a Cochran's Q with a significance level of < 0.05 (based on chi‐square distribution), and *I* ^2^ values were used to judge the degree of heterogeneity. *I* ^2^ values of 25%, 50%, and 75% indicate small, medium, and large amounts of heterogeneity, respectively [50].

**TABLE 1:** Experimental characteristics of the studies included in the qualitative synthesis.

Study (year)	*N*	Age	Motor task	Level	Session	Training target	Integrating	Personalized feedback	Feedback type	Type of control group
[8]	24	/	Archery	Expert	45–75 min*1	Single (low potential)	I	GI	Visual	Non‐any
[36]	18	20.67	Dance	Novice	20 min*10	Single (*α*/*θ*)	UI	SI	Audio	Non‐any
[37]	24	21.96	Archery	Expert	20 min*12	Multiple (SMR, *θ*, *β*)	UI	GI	Both	Non‐any
[38]	24	30.45	Rifle shooting	Expert	60 min*15	Multiple (SMR, *β*)	UI	GI	Both	Sham
[39]	28	/	Videomotor	Expert	15	Single (*α*)	UI	GI	/	/
[4, 5, 6]	64	/	Dance	Expert	20 min*10	Single (*α*/*θ*)	UI	SI	Audio	Non‐any
[7, 17]	16	21.45	Golf putting	Expert	30 min*8	single (SMR)	I	SI	Both	Mock
[21]	24	22.00	Golf putting	Expert	60 min*3	Multiple (high *α*, *θ*)	I	GI	Audio	Sham
[40]	21	20	(Soccer & sprint hurdle)	Expert	30 min*15	Single (*α*)	UI	GI	Audio	Biofeedback
[41]	16	21.00	Dynamic balance	Expert	25mims*15	Multiple (*θ*, *β*)	UI	GI	Both	Sham
[42]	40	27	Cycling TTE test	Novice	17 min*1	Multiple (left *α*, right *α*)	UI	GI	Both	Watch video clips
[11]	36	36.14	Golf putting	Expert	1	Single (FMT)	I	SI	Audio	Sham
[43]	32	21.1	Golf putting	Novice	20 min*6	Single (SMR)	UI	GI	Both	Sham
[44]	40	25.4	Golf putting	Novice	20 min*6	Single (SMR)	I	GI	Both	Sham
[45]	28	21.7	Rifle shooting performance	Expert	30 min*6	Single (FMT)	I	GI	Audio	Non‐any
[10]	30	27.45	Golf putting	Novice	30–45 min*1	Single (Mu)	I	GI	Audio	Sham
[9]	36	27.4	Golf putting	Novice	1	Single (Mu)	I	SI	Audio	Sham
[12]	44	26.83	Golf putting	Expert	1	Single (SMR)	I	SI	Audio	Non‐any
[46]	17	24.63	Golf	Expert	1	Single (SMR)	I	SI	Audio	Non‐any
[47]	20	25.5	Air pistol	Expert	16	Single (T3 alpha)	I	SI	Both	Non‐any
[48]	16	25.5	Pistol shooting	Expert	30 min*20	Multiple (T3–T4, T4–P4)	UI	SI	Both	Non‐any

*Note:* * = multiplication, meaning (minutes) * (sessions).

Abbreviations: GI, general instruction; I, integrated; *N*, Sample size; SI, specific instruction; UI, unintegrated.

In the subgroup analyses, we examined potential moderating variables identified in prior research that may have influenced the effects of neurofeedback [11, 13, 15, 16, 25].

First, to rigorously assess the quality of previous research, we incorporated part of the CRED‐nf checklist (the “essential” checklist items) for methodological evaluation. These items are fundamental requirements that must be adhered to in neurofeedback research. Failure to meet these items may compromise the validity and reliability of the study, while the remaining items are classified as merely “encouraged” checklist items.

Based on the median score, CRED‐nf scale scores were classified as higher or lower than median scores. Second, factors such as participant expertise (novice vs. expert), integration of neurofeedback into the sport task and provision of personalized feedback have not been explored in prior meta‐analytic investigations. Specifically, the analyses were conducted for the following five categorical items:

1. Level, the participants are either experts or novices [23].
2. Integrating, whether neurofeedback training was integrated into the sport motor task [15, 16, 24]. For example, if neurofeedback training was conducted simultaneously with a sport motor task, such as shooting a basketball or putting, it would be considered integrated. In contrast, if the training consisted solely of neurofeedback sessions without any concurrent motor task, it would be classified as non‐integrated.
3. Sessions, number of training sessions, categorized as short (1–3), middle (4–8), or long (9+) [13].
4. Types, whether the sport was a precision‐based type [25].
5. Training target, quantity of training objectives, categorized as single or multiple [25].
6. Personalized feedback, whether personalized feedback was provided [11]. For example, if the feedback was tailored to each individual's performance, such as providing specific guidance on how to adjust their mental state, it would be considered specific instruction. In contrast, if all participants received the same standardized feedback without customization, it would be classified as general instruction.

By conducting these subgroup analyses, we were able to explore potential moderating factors that may have contributed to the heterogeneity in findings and further elucidate the role of neurofeedback training in improving sport performance. Statistical significance was determined for all tests at a *p* value less than 0.05. All data were combined using Stata18.0 [35]. https://www.stata.com/features/overview/meta‐analysis/.

## 3 Results

### 3.1 Search Selection

Initially, a total of 3602 articles were obtained from various databases. After screening the titles and abstracts for relevance, 3570 articles were excluded from further analysis. They were excluded due to various reasons: language discrepancy (*n* = 24); reviews or comments (*n* = 1972); without healthy participants or NFT (*n* = 1346); no outcome of sport performance (*n* = 216); no experiment design (*n* = 12). Subsequently, a full‐text eligibility assessment was conducted on the remaining 27 articles. Among these, 5 articles were excluded due to the lack of a control group. The full texts of the remaining 25 studies were included for qualitative synthesis. Finally, out of these 25 studies, 21 studies met the eligibility criteria for the meta‐analysis. The reasons for exclusion in the meta‐analysis phase were as follows: insufficient reported results for effect size calculation (*n* = 4). The indicated funding of the respective included studies does not raise suspicion of any kind. A flow diagram of the literature search is displayed in Figure 1.

### 3.2 Study Characteristics and Narrative Synthesis

Table 1 summarizes the key characteristics of 25 studies. The publication years of these studies ranged from 1991 to 2025, with more than half published after 2017. The data presented in this section encompasses a diverse range of studies investigating various motor tasks and outcome measures. The feedback provided to participants, derived from targeted brain activity, was presented in visual, auditory, or audiovisual formats. In EEG‐based NFT, the frequency bands used included theta (4–7.5 Hz), alpha (8–13 Hz), sensorimotor rhythm (SMR), (12–15 Hz), and beta (15–30 Hz). Electrodes placed around the sensorimotor cortex (C3, Cz, C4) and midline parietal cortex (Pz) were commonly used [9, 10, 17, 51, 52]. Alpha rhythm and SMR were frequently employed individually, while theta and beta rhythms were often combined, such as in the theta/beta ratio [21, 41, 43]. Specific electrode placements were associated with distinct frequency bands; for instance, SMR from the CZ channel and theta band activity from the PZ channel were frequently utilized in NFT.

The intervention periods ranged from 30 min in a single session to 900 min delivered across 15 sessions over a period of 4.5 months. Regarding control conditions, 9 studies employed sham feedback, 3 studies used different NFT protocols targeting alternate EEG frequency bands, 5 studies conducted general training without neurofeedback (e.g., mental practice), and 12 studies employed a control condition in which participants remained at rest without any intervention. Regarding motor performance measures, the majority of research concentrated on precision movements, including archery, shooting, dart throwing, and golf putting. Other studies emphasized dance performance, ideomotor actions, dynamic balance, cycling endurance, ice hockey shooting, and basketball performance.

### 3.3 Risk of bias in Studies

**FIGURE 2:**
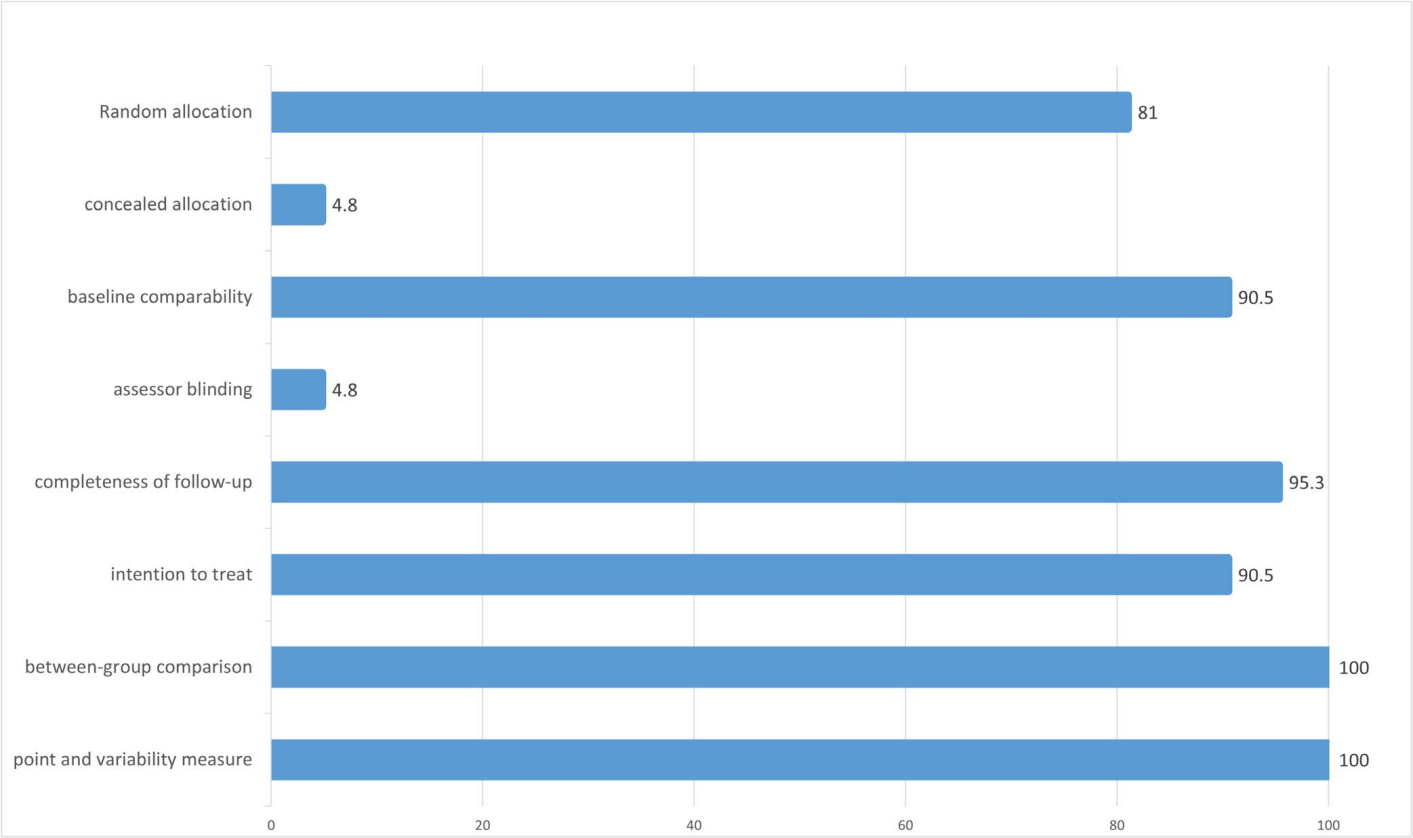
Study quality overview.

Figure 2 presents an overview of the study quality included in the review. None of the 22 studies included in the analysis met the criteria to be categorized as low risk of bias in all assessed domains. Regarding the random allocation, the majority of studies (81%) offered information on the randomization process used to assign participants to each group. Additionally, 2 studies did not report matching baseline levels of sports performance between the groups before neurofeedback training (9.5%). In terms of allocation concealment, only one study [12], provided sufficient information on the allocation method and was classified as low risk, while the remaining studies had an unclear risk. Only one study used a double‐blind design [41]. Regarding attrition bias, one study was categorized as high risk because fewer than 85% of participants initially assigned to each group completed at least one primary outcome measure [36]. Reporting bias was deemed unclear for the great majority of studies, as none of them had pre‐registered protocols. Finally, regarding other possible sources of bias, nine studies that did not report conflicts of interest (COI) or the method used for determining sample size were assessed as having unclear risks. (see Appendix S3).

### 3.4 The CRED‐Nf Checklist

Table 2 presents a chart created based on the CRED‐nf checklist, categorizing all included literature according to whether it met the checklist criteria. Among these, there were five items with considerable inconsistency in quality across all documents, namely, 1. report whether participants were provided with a strategy (3b), 2. report methods used for online data processing and artifact correction (3d), 3. report and justify the reinforcement schedule (4b), 4. report neurofeedback regulation success based on the feedback signal (5a), and 5. run correlational analyses between regulation success and behavioral outcomes (6b).

**TABLE 2:** The CRED‐nf checklist [22].

ID	1b	2a	2b	3b	3d	4a	4b	4c	4d	4e	5a	5b	5c	6a	6b	Total
[8]	0	1	0	0	1	1	1	1	0	0	0	0	1	1	0	7
[36]	0	1	0	1	0	1	1	1	0	1	1	0	1	1	1	10
[37]	0	1	0	1	0	1	0	1	1	1	0	0	1	1	1	9
[38]	0	1	0	1	0	1	1	1	0	1	0	0	1	1	0	8
[39]	0	1	0	0	0	0	0	1	0	1	1	0	1	1	0	6
[4, 5, 6]	0	1	0	1	1	1	1	1	1	1	1	1	1	1	1	13
[7, 17]	0	1	0	1	1	1	1	1	1	1	1	1	1	1	0	12
[21]	0	1	0	1	1	1	1	1	1	1	0	1	1	1	0	11
[40]	0	1	0	0	0	1	1	1	0	1	0	0	1	1	0	7
[41]	0	1	1	0	1	1	0	1	1	1	0	0	1	1	0	9
[42]	1	1	0	1	1	1	1	1	1	1	1	1	1	1	0	13
[11]	1	1	0	1	1	1	1	1	1	1	1	0	1	1	1	13
[43]	1	1	0	1	1	1	1	1	1	1	1	1	1	1	1	14
[44]	1	1	0	1	1	1	1	1	1	1	1	1	1	1	1	14
[45]	1	1	0	1	1	1	1	1	0	1	1	1	1	1	0	12
[10]	1	1	0	1	1	1	1	1	1	1	1	0	1	1	1	13
[9]	1	1	0	1	1	1	1	1	1	1	1	0	1	1	1	13
[12]	1	1	0	1	1	1	1	1	1	1	1	0	1	1	1	13
[46]	0	1	0	1	1	1	1	1	1	1	1	1	1	1	0	13
[47]	0	1	0	1	1	1	1	1	1	1	1	1	1	1	1	13
[48]	1	1	0	1	0	1	1	1	1	1	1	0	1	1	0	11
Score (%)	42.9	100	4.8	81	74.1	90.5	85.7	100	74.1	90.5	74.1	42.9	100	100	47.6	

1b. Justify sample size 2a. Employ control group(s) or control condition(s) 2b. Double‐blind 3b. Report whether participants were provided with a strategy 3d. Report methods used for online‐data processing and artifact correction 4a. Report how the online‐feature extraction was defined 4b. Report and justify the reinforcement schedule 4c. Report the feedback modality and content 4d. Collect and report all brain activity variable(s) and/or contrasts used for feedback, as displayed to experimental participants 4e. Report the hardware and software used 5a. Report neurofeedback regulation success based on the feedback signal 5b. Plot within‐session and between‐session regulation blocks of feedback variable(s), as well as pre‐to‐post resting baselines or contrasts 5c. Statistically compare the experimental condition/group to the control condition(s)/group(s) (not only each group to baseline measures) 6a. Include measures of clinical or behavioral significance, defined a priori, and describe whether they were reached 6b. Run correlational analyses between regulation success and behavioral outcomes

### 3.5 Results of Syntheses

**FIGURE 3:**
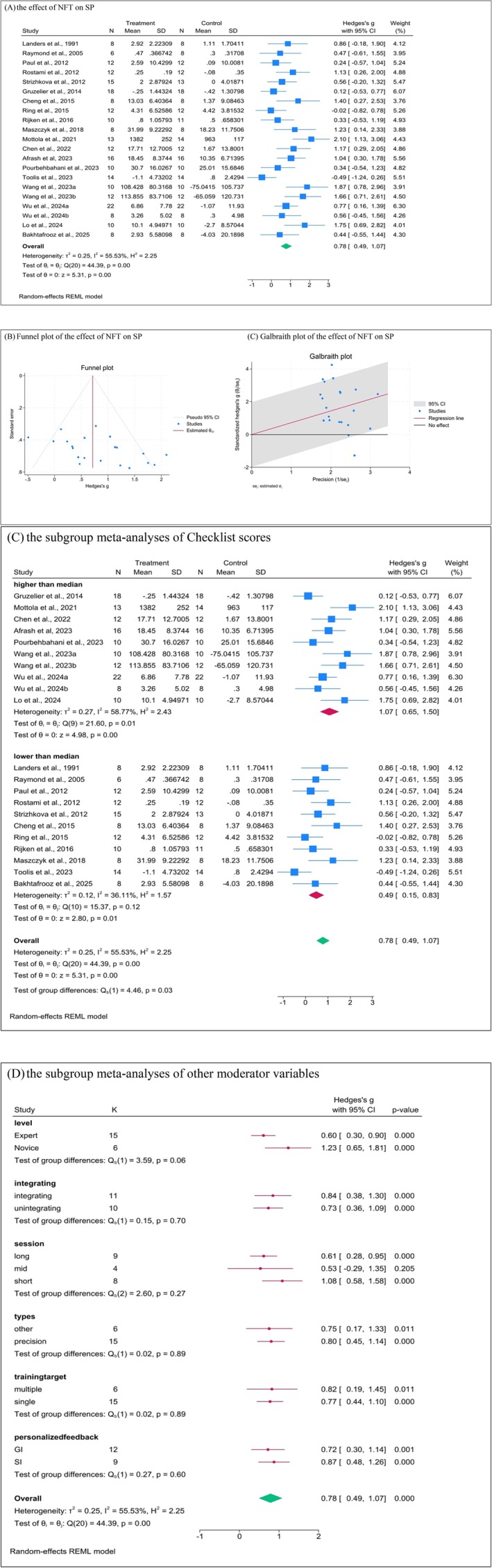
Forrest plot of the effect of NFT on SP.

The meta‐analysis incorporated 21 studies, involving a total of 271 participants. The analysis revealed that neurofeedback training (NFT) had a moderate impact on sports motor tasks, with a Hedges's g of 0.78% and a 95% confidence interval (CI) of 0.49–1.07. This result was statistically significant (*Z* = 5.31, *p* = 0.00) and exhibited medium‐level heterogeneity (*I* ^2^ = 55.53%). This meta‐analysis revealed a positive effect of EEG neurofeedback compared to the control group from pre‐test to post‐test. The forest plots of the effects of NFT on sports motor tasks are presented in Figure 3A.

To assess the presence of publication bias among the included studies, the results did not suggest the publication bias across all outcomes, as evidenced by Rosenthal's fail‐safe N and Egger's test. The funnel plot depicting the effect of neurofeedback training on sports performance, as shown in Figure 3B,C, did not reveal any obvious asymmetry indicative of publication bias.

Further analysis through subgroup meta‐analyses examined the influence of methodological quality, specifically scores derived from the CRED‐nf checklist, on the efficacy of NFT.

The NFT appeared to have a stronger impact in the “higher than median” group, with a statistically significant difference in effect sizes between the two groups (Q(1) = 4.46, *p* = 0.03). This shows that the effect sizes between the “higher than median” group (Hedges's g = 1.07, 95% CI [0.65, 1.50]) and the “lower than median” group (Hedges's g = 0.49, 95% CI [0.15, 0.83]) were significantly different. Additionally, in the higher than median group, there is significant and moderate heterogeneity (*I* ^2^ = 58.77%), indicating variability in the effect sizes across studies within this group. In contrast, the lower than median group showed only low heterogeneity (*I* ^2^ = 36.11%), suggesting more consistent effect sizes across the studies in this group. The forest plots of the subgroup meta‐analyses of NFT on sports performance are presented in Figure 3C.

Subgroup meta‐analyses were conducted for categorical items in Figure 3D. Among all the moderating factors, only the participants level of expertise (experts or novices) showed a nearly significant difference (Q(1) =3.59, *p* = 0.06). For experts, the effect size was moderate, with g = 0.60 and a 95% CI [0.30, 0.90]. This suggests that interventions had a medium effect on experts. For novices, the effect size was larger, with g = 1.23 and a 95% CI [0.65, 1.81]. This means that while novices showed a larger effect size, the difference between the two groups is not strong enough to conclude that the level of expertise significantly moderates the effect of interventions.

For the other categorical moderators examined, such as the number of training sessions, whether the training was integrated, the types of performance measures, the number of training objectives, and the provision of personalized feedback, none of these factors exhibited a significant moderating effect (all Q(1) < 2.6, all *p* > 0.27).

## 4 Discussion

The purpose of this study was to conduct a systematic review and meta‐analysis, incorporating the CRED‐nf checklist for evaluating the effects of NFT on sports performance and identifying key methodological factors. The main findings of this study indicate that EEG NFT has a moderate positive impact on sports performance. The NFT appeared to have a stronger effect in cases rated “higher than median” according to the CRED‐nf checklist. Additionally, the participants' level showed a nearly significant moderating effect, while the integration of NFT into the sport task did not reveal any moderating effects.

This meta‐analysis delineated a moderate positive impact of EEG NFT on sport performance, with a Hedges's g of 0.78 and a 95% CI of 0.49–1.07. These results corroborate the findings of prior meta‐analyses, which presented a diverse range of outcomes concerning the efficacy of EEG NFT in improving sports performance [13, 14]. Previous meta‐analyses on neurofeedback did not solely focus on sports performance, and many high‐quality articles published in recent years have not been included in the analysis due to the rapid development of neurofeedback. Compared to the previous two meta‐analyses ([14], 80%; [13], 53%), this study was 100% focused on motor performance outcomes. Additionally, only 38% [13] and 50% [14] of the included literature overlapped with this study.

Consequently, it updated the existing published research and identified the impact on sports performance. This study also underscores the necessity for standardized and rigorous methodologies in future neurofeedback research to optimize sports performance outcomes. The following sections will discuss the quality of NFT interventions, the participants' levels, the impact of integration on NFT efficacy in sports, the effects of training session numbers on NFT outcomes, other moderator variables, limitations, and recommendations for future studies.

### 4.1 Quality of NFT Interventions

The subgroup analyses based on the CRED‐nf checklist [22] highlighted a significantly stronger effect in the “higher than median” group (Hedges's g = 1.07) compared to the “lower than median” group (Hedges's g = 0.49), with a statistically significant difference in effect sizes (Q(1) = 4.46, *p* = 0.03). Although the “higher than median” studies exhibited greater heterogeneity, this distinction offers a plausible explanation for the inconsistent results observed in prior reviews. It suggested that the varying levels of methodological rigor across studies contributed to the heterogeneity of findings [15, 16]. Among the high‐CRED score studies, the majority reported or implemented the following: (1) participant strategies, (2) online data processing and artifact correction methods, (3) reinforcement schedules, (4) neurofeedback regulation success, and (5) correlational analyses between regulation success and behavioral outcomes [10, 11, 12, 43]. These factors may contribute to the enhanced efficacy of neurofeedback observed in these relatively higher‐quality studies. In contrast, studies in the “lower than median” group more frequently lacked justification for sample size and sufficient reporting or justification of the reinforcement schedule. These may partially explain the weaker effects observed in this group [22]. However, it is important to note that categorizing studies into only two groups based on a median split is a relatively simplistic approach. This method may overlook more nuanced differences in methodological rigor and fail to capture the full spectrum of quality across studies.

### 4.2 The Participants' Level

The participants' level may serve as a moderating variable that explains the varied findings in this review. Differences in performance outcomes may arise depending on whether the participants are experts or novices. Some studies have recruited professional‐level athletes [12], pre‐elite players [7, 8, 17], university level players [37], or novices [9, 53]. The disparities in participant skill levels could lead to confounding the effects of neurofeedback training. Although some studies have reported the players' training experience or competitive level, there are still discrepancies when making comparisons across different sports domains [23]. A potential solution is to use quantitative metrics like handicap scores, as in golf, to better estimate and compare the skill levels of participants across studies [54]. This approach may provide a more standardized way to account for individual differences in expertise and explore the relationship between skill level and the efficacy of neurofeedback interventions.

### 4.3 Impact of Integrating Neurofeedback Training Into Sports Task

The comparison between integrating and non‐integrating NFT interventions revealed no significant differences (Q(1) = 0.15, *p* = 0.70). Prior research, including studies by Gruzelier et al. [55] and Renshaw et al. [24], has highlighted the potential benefits of integrating NFT into sport tasks, such as facilitating skill transfer and enhancing performance. This approach facilitates participants' understanding of specific motor images or strategies necessary to modify their EEG patterns to meet training thresholds. However, the subgroup analysis in our current study did not support this finding—the moderator effect was non‐significant between the integrated and non‐integrated neurofeedback training approaches. This discrepancy may stem from the varied training instructions and diverse protocols used in previous NFT studies in sports [56]. To our knowledge, there are a limited number of studies directly examining the differences in integrating NFT into sports tasks. These considerations underscore the importance of adopting a standardized checklist, for example, the CRED‐nf checklist, to advance the field by conducting replicable and methodologically sound studies. Lastly, further empirical evidence is necessary to conclusively determine the impact of integrating neurofeedback training into sports tasks. This concept is supported by a review by Mirifar et al. [15, 16], which suggested that the effectiveness of NFT might be enhanced when training is closely integrated with the target sports task.

### 4.4 Effects of the Training Session Number on NFT Outcomes

In the subgroup meta‐analyses, the number of training sessions emerged as no significant differences (Q(1) =2.6, *p* = 0.27); studies using 3–8 sessions were not significant and exhibited higher variability (*n* = 4; g = 0.53, 95% CI = −0.29–1.35; *p* = 0.21). The studies using more than 8 sessions demonstrated moderate effect sizes (*n* = 9; g = 0.61, 95% CI = 0.28–0.95; *p* < 0.001), and the studies using fewer than 3 sessions demonstrated larger effect sizes (*n* = 8; g = 1.08, 95% CI = 0.58–1.58; *p* < 0.001). These fewer than 3 sessions studies primarily recently utilized integrated neurofeedback training approaches. These studies tended to provide single‐target feedback in a single session and focused on precision‐based sports [8, 9, 10, 11, 12, 21]. This finding suggests that the optimal dose of NFT may depend on the specific mechanisms underlying the training effects, which may differ for shorter versus longer interventions [57]. NFT lasting longer than 1 week and totaling more than 125 min of cumulative training time would be recommended for motor performance improvements. However, these results may not be entirely consistent with previous meta‐analyses, which indicated that sessions typically lasting 20–30 min, conducted 2–3 times per week, and extending from a minimum of 5–10 sessions to an optimal range of up to 40 sessions are necessary for significant neuroplastic changes [13, 14, 15, 16, 56]. It is recommended that NFT for motor performance improvements should last longer than 1 week and exceed 125 min of cumulative training time [13]. The effects of NFT can be long‐lasting, potentially enduring for months, although periodic maintenance sessions may be necessary to sustain benefits. Notably, the current meta‐analysis includes more single‐session studies than previous meta‐analyses, which may influence the comparative results.

### 4.5 Other Moderator Variables

As for the other moderator variables, such as the types of performance measures, the number of training objectives, and the provision of personalized feedback, none of these factors exhibited a significant moderating effect (all Q(1) < 2.6, all *p* > 0.27). These findings are inconsistent with the results of past review studies [13, 15, 16, 25].

Previous research has not provided participants with a specific strategy (i.e., how to regulate a certain brain region) in their verbal instructions to achieve an optimal mental state [20, 58]. This lack of guidance may have imposed extra cognitive demands or been insufficient to elicit changes in EEG power, as participants may have been puzzled or uncertain about what to do in neurofeedback training (Munoz‐Moldes & Cleeremans, 2020). It is worth noting that Chen et al. [11] demonstrated that their verbal instructions were function‐directed. The rationale is that verbal instructions should be directly related to the features of brain function for achieving an optimal mental state [58, 59]. Function‐directed instructions in verbal instructions can avoid target‐irrelevant brain activity or a slower timescale in the initial learning phases [58], because they explicitly guide individuals to enter a specific mental state. In summary, providing function‐directed verbal instructions in neurofeedback training may be essential for guiding participants effectively and optimizing their mental states.

This study extended previous meta‐analyses that only included studies which used cognitive test performance or questionnaire scores as well as studies with no control group or those with uncontrolled neurofeedback settings. In addition, this study reaffirms the potential of EEG NFT and enhances our understanding of its role in sports psychology through a rigorous, evidence‐based, and manipulation‐checked approach. Notably, the current meta‐analysis also accounted for the substantial variability in methodological quality as assessed by checklist scores and focused specifically on sports performance tasks across the included studies, which has been a key limitation identified in prior reviews. Although the range of moderator factors has been expanded, several issues still warrant further discussion. For example, the psychological states following NFT training have been less frequently reported in the literature. A recent study by [12], demonstrated that in the SMR NFT condition, participants exhibited decreased attentional engagement, decreased conscious motor control, and increased relaxation in putting tasks compared to the control condition. In the theoretical framework of sports psychology, optimal sports performance is characterized by effortless attention and automatic, nearly effortless experiences [60]. This supports the core concept of the psychomotor efficiency hypothesis [61, 62], which emphasizes selective and specific neural processing toward the motor task [51], aligning with the autonomous stage in motor learning [63]. Therefore, the psychological states associated with NFT training are also worthy of further exploration in future research.

Several limitations of the current meta‐analysis warrant discussion. The included studies exhibited significant variability in sample sizes, participant characteristics, neurofeedback protocols, and outcome measures, complicating direct comparisons and reducing the generalizability of the findings. Despite using the CRED‐nf checklist to assess study quality, many included studies had methodological limitations. These limitations included inadequate blinding, lack of randomization, small sample sizes, failure to classify the impact of EEG neurofeedback training on different skill levels, and inconsistent control group strategies. These issues may introduce bias and affect the reliability of the results. Additionally, most included studies focused on short‐term interventions, often comprising fewer than three sessions (seven studies, 39% of included studies), leaving the long‐term effects and sustainability of neurofeedback training for sports performance unclear. The meta‐analysis primarily considered motor performance outcomes, potentially overlooking other relevant aspects of sports performance, such as cognitive functions, emotional regulation, and physiological responses. Additionally, differences in neurofeedback protocols, including electrode placements, targeted frequency bands, and feedback modalities, contribute to inconsistent results, highlighting the need for standardization in protocol design for more conclusive outcomes. Moreover, Using the median to classify study quality is a relatively crude approach. It would be good for future research to revisit this approach when there is a greater body of literature to more clearly dichotomize high and low quality papers.

Future EEG NFT studies aimed at enhancing sports performance should address the methodological limitations identified in this review. First, training protocols and target brain regions must be grounded in established theoretical frameworks and empirical evidence linking specific neural signatures to sports‐relevant cognitive and motor processes [64, 65, 66]. Second, research should prioritize understanding the long‐term effects of NFT and investigate retention effects to evaluate the actual impact on sports performance enhancement [44], thereby increasing its practical applicability. Third, researchers should focus on specific outcome measures such as speed, accuracy, and dexterity to determine which aspects of motor performance benefit most from NFT. Fourth, comparing NFT effects with sham feedback control groups is essential to accurately assess the true effects and eliminate placebo influences. Fifth, NFT interventions should be meticulously designed with clear, step‐by‐step procedures, considering athletes' skill levels and the unique demands of the target sport [55]. This approach ensures that neurofeedback training is optimally tailored to athletes' needs and specific sports contexts, enhancing the efficacy and real‐world applicability of these interventions [56, 67, 68]. Lastly, further research is needed to determine the optimal training duration and frequency for different sports and athlete populations.

## 5 Conclusions

The present systematic review and meta‐analysis offers a comprehensive evaluation of the effects of neurofeedback training (NFT) on sports performance. The results indicate that NFT can have a moderate positive impact on various motor skills and sports‐related outcomes, with an overall Hedges's g of 0.78 (95% CI: 0.49–1.07, *p* = 0.00). This finding aligns with previous meta‐analyses [13, 14], while expanding the evidence base by including a larger number of more recently published studies that specifically focused on sports performance. Importantly, the subgroup analyses revealed that the quality of the NFT studies, as evaluated by the CRED‐nf checklist, significantly moderator the intervention's effectiveness. In conclusion, NFT showcases a moderate positive impact on sports performance, particularly when high‐quality methodologies are employed, according to the CRED‐nf checklist, underscoring the importance of rigorous study designs in future research. Future research should focus on developing well‐grounded and clearly specified NFT tailored to the unique needs of athletes and sports, while also addressing the methodological limitations identified in this review.

## Author Contributions

**Chien‐Lin Yu:** writing – original draft, visualization, project administration. **Ming‐Yang Cheng:** writing – original draft, data curation, conceptualization. **Xin An:** writing – review and editing, data curation, conceptualization. **Ting‐Yu Chueh:** writing – review and editing, project administration, data curation. **Jia‐Hao Wu:** writing – review and editing, data curation, methodology, formal analysis. **Kuo‐Pin Wang:** writing – review and editing, supervision, funding acquisition, conceptualization. **Tsung‐Min Hung:** writing – review and editing, supervision, funding acquisition, conceptualization.

## Conflicts of Interest

The authors declare no conflicts of interest.

## Supporting information


Data S1.

## Data Availability

Data available on request from the authors. The data that support the findings of this study are available from the corresponding author upon reasonable request.
