# Supplementary material for: The Effect of EEG Neurofeedback Training on Sport Performance: A Systematic Review and Meta‐Analysis
Source: Scand J Med Sci Sports. 2025 Apr 24;35(5):e70055. doi: 10.1111/sms.70055 (PMC12019780; doi:10.1111/sms.70055)
Supplement: Supplementary file 1 — Data S1. [file SMS-35-e70055-s001.docx]

Supplementary Appendix S1. Data source & Key words

| PubMed  (neurofeedback[Title/Abstract] OR neuro-feedback[Title/Abstract] OR EEG-biofeedback[Title/Abstract] OR EEG-feedback[Title/Abstract] OR electrocortical biofeedback[Title/Abstract] OR NFB training[Title/Abstract]) AND (sport*[Title/Abstract] OR elite*[Title/Abstract] OR athlete*[Title/Abstract] OR exercise[Title/Abstract] OR player*[Title/Abstract] OR performance*[Title/Abstract] OR skill*[Title/Abstract] OR archer*[Title/Abstract]) AND (RCT[Title/Abstract] OR control[Title/Abstract] OR groups[Title/Abstract]) NOT (disease[Title/Abstract] OR disorder[Title/Abstract] OR injury[Title/Abstract]) | Scopus  ( TITLE-ABS-KEY ( "*neurofeedback" OR "neuro-feedback" OR "EEG-biofeedback" OR "EEG-feedback" OR "electrocortical biofeedback"OR "NFB training" ) AND TITLE-ABS-KEY ( "sport*" OR "elite*" OR "athlete*" OR "exercise" OR "player*" OR "performance*" OR "skill*" OR "archer*" ) AND TITLE-ABS-KEY ( "RCT" OR "control" OR "groups" ) AND NOT TITLE-ABS-KEY ( ''disease'' OR ''disorder'' OR ''injury'' ) ) | Web of science (WOS)  (TS=("neurofeedback" OR "neuro-feedback" OR "EEG-biofeedback" OR "EEG-feedback" OR "electrocortical biofeedback" OR "NFB training") AND TS=("sports" OR "elite*" OR "athlete*" OR "exercise" OR "player*" OR "performance*" OR "skills" OR "archers") | EBSCO host  (TX=("neurofeedback" OR "neuro-feedback" OR "EEG-biofeedback" OR "EEG-feedback" OR "electrocortical biofeedback" OR "NFB training") AND TX=("sports" OR "elite*" OR "athlete*" OR "exercise" OR "player*" OR "performance*" OR "skills" OR "archers") NOT TX= (''disease'' OR ''disorder'' OR ''injury'') |
| --- | --- | --- | --- |

Supplementary Appendix S2. The CRED-nf checklist


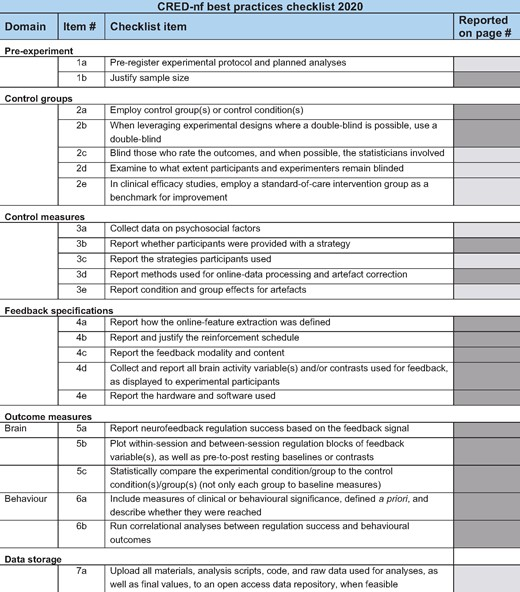
**Supplementary Appendix S3.** Quality assessment of included studies

| item | Random allocation | concealed allocation | baseline comparability | assessor blinding | completeness of follow-up | intention to treat | between-group comparison | point and variability measure |
| --- | --- | --- | --- | --- | --- | --- | --- | --- |
| Landers et al., 1991 | 1 | 0 | 1 | 0 | 1 | 1 | 1 | 1 |
| Raymond et al., 2005 | 1 | 0 | 1 | 0 | 0 | 0 | 1 | 1 |
| Paul et al., 2012 | 1 | 0 | 1 | 0 | 1 | 1 | 1 | 1 |
| Rostami et al., 2012 | 0 | 0 | 1 | 0 | 1 | 1 | 1 | 1 |
| Strizhkova et al., 2012 | 0 | 0 | 1 | 0 | 1 | 1 | 1 | 1 |
| Gruzelier et al., 2014 | 1 | 0 | 1 | 0 | 1 | 1 | 1 | 1 |
| Cheng et al., 2015 | 1 | 0 | 1 | 0 | 1 | 1 | 1 | 1 |
| Ring et al., 2015 | 1 | 0 | 1 | 0 | 1 | 1 | 1 | 1 |
| Rijken et al., 2016 | 0 | 0 | 0 | 0 | 1 | 0 | 1 | 1 |
| Maszczyk et al., 2018 | 1 | 0 | 1 | 1 | 1 | 1 | 1 | 1 |
| Mottola et al., 2021 | 1 | 0 | 1 | 0 | 1 | 1 | 1 | 1 |
| Chen et al., 2022 | 0 | 0 | 1 | 0 | 1 | 1 | 1 | 1 |
| Afrash et al, 2023 | 1 | 0 | 1 | 0 | 1 | 1 | 1 | 1 |
| Pourbehbahani et al., 2023 | 1 | 0 | 1 | 0 | 1 | 1 | 1 | 1 |
| Toolis et al., 2023 | 1 | 0 | 1 | 0 | 1 | 1 | 1 | 1 |
| Wang et al., 2023a | 1 | 0 | 1 | 0 | 1 | 1 | 1 | 1 |
| Wang et al., 2023b | 1 | 0 | 1 | 0 | 1 | 1 | 1 | 1 |
| Wu et al., 2024a | 1 | 1 | 0 | 0 | 1 | 1 | 1 | 1 |
| Wu et al., 2024b | 1 | 0 | 1 | 0 | 1 | 1 | 1 | 1 |
| Lo et al., 2024 | 1 | 0 | 1 | 0 | 1 | 1 | 1 | 1 |
| Bakhtafrooz et al., 2025 | 1 | 0 | 1 | 0 | 1 | 1 | 1 | 1 |
| Score (%) | 81 | 4.8 | 90.5 | 4.8 | 95.3 | 90.5 | 100 | 100 |

Note. PEDro scoring: Yes = 1, No = 0, NR = 0; Item 1 (eligibility criteria and source) is not used to calculate the PEDro score; the following cut-points were used to describe the quality of papers: 9-10 (excellent), 6-8 (good), 4-5 (fair), ≤3 (poor)
